# Hydroxyurea Induces Bone Marrow Mesenchymal Stromal Cells Senescence and Modifies Cell Functionality In Vitro

**DOI:** 10.3390/jpm11111048

**Published:** 2021-10-20

**Authors:** Sunčica Kapor, Milica Vukotić, Tijana Subotički, Dragoslava Đikić, Olivera Mitrović Ajtić, Milica Radojković, Vladan P. Čokić, Juan F. Santibanez

**Affiliations:** 1 Clinical Hospital Center “Dr Dragiša Mišović-Dedinje”, Department of Hematology, University of Belgrade, 11000 Belgrade, Serbia; suncicabjelica@gmail.com (S.K.); milicaradojkovic1@gmail.com (M.R.); 2 Group for Molecular Oncology, Institute for Medical Research, National Institute of Republic of Serbia, University of Belgrade, 11129 Belgrade, Serbia; milica.tosic@imi.bg.ac.rs (M.V.); tijana@imi.bg.ac.rs (T.S.); dragoslava@imi.bg.ac.rs (D.Đ.); oliveram@imi.bg.ac.rs (O.M.A.); vl@imi.bg.ac.rs (V.P.Č.); 3 Faculty of Medicine, University of Belgrade, 11000 Belgrade, Serbia; 4 Centro Integrativo de Biología y Química Aplicada (CIBQA), Universidad Bernardo O’Higgins, General Gana 1780, Santiago 8370854, Chile

**Keywords:** hydroxyurea, bone marrow mesenchymal stem cells, senescence, differentiation, immunosuppression

## Abstract

Hydroxyurea (HU) is an antineoplastic agent that functions as an antimetabolite compound by inhibiting the ribonucleotide reductase. HU acts mainly as a cytostatic drug that through DNA replication stress may trigger a premature senescence-like cell phenotype, though its influence on bone marrow-derived mesenchymal stem/stromal cell (BMMSC) functions has not elucidated yet. Our results indicate that HU inhibits the growth of human BMMSC alongside senescence-like changes in both morphology and replicative potential, provokes cell cycle arrest at the S phase without affecting cellular viability and induces the expression of senescence-associated β-galactosidase and p16INK4. Moreover, HU-induced senescent BMMSC, although they did not change MSC markers expression, exhibited reduced capacity osteogenic and adipogenic differentiation. Conversely, HU treatment increased immunoregulatory functions of BMMSC compared with untreated cells and determined by T-cell proliferation. Interestingly, HU did not influence the capacity of BMMSC to induce monocytic myeloid-derived suppressor cells. Thus, these results suggest that HU improves the BMMSC functions on the T-cell inhibition and preserves their interaction with myeloid cell compartment. Mechanistically, BMMSC under HU treatment displayed a downregulation of mTOR and p38 MAPK signaling that may explain the reduced cell differentiation and increased immunomodulation activities. Together, the results obtained in this investigation suggest that HU by inducing senescence-like phenotype of BMMSC influences their cellular differentiation and immunoregulatory functions.

## 1. Introduction

Hydroxyurea (HU, also named hydroxycarbamide) is non-alkylating antineoplastic and antiviral drug, which functions as an antimetabolite compound due to its inhibitory function on ribonucleotide reductase (RNR). Inhibition of DNA replication by HU is reversible, indicating that the drug is likely a cytostatic agent [1]. This agent has been used to treat neoplastic diseases and non-neoplastic conditions such as infectious and dermatology diseases [1]. Because of all the positive effects of HU therapies, it is nominated as an “essential medicine” by the World Health Organization [2]. Moreover, hydroxyurea is a first-line cytoreduction agent in high-risk patients with a myeloproliferative neoplasm [3].

Mesenchymal stromal/stem cells (MSCs) are crucial elements of the bone marrow (BM) niche where they provide newly formed osteoblasts for bone tissue regeneration and tightly control hematopoietic stem cells (HSCs) fate [4,5]. They are characterized by the expression of surface markers CD73, CD90, and CD105, and lack of hematopoietic lineage markers expression [6,7]. Furthermore, MSCs are also characterized by expressing low MHC class I and no MHC class II and costimulatory molecules CD40, CD80, and CD86 expression, thus preventing alloreactive antibody production and their elimination by the immune system after transplantation [8].

MSCs are multipotent cells characterized by their ability to differentiate into adipocytes, chondrocytes, and osteoblasts [4,5]. Besides, MSC differentiation is tightly regulated by several intracellular signaling that drive the fate of differentiation [9]. In this sense, the mammalian target of rapamycin (mTOR), a member of the phosphatidylinositol 3-kinase-related kinase family of protein kinases, regulates MSC committed and critically influences osteogenic and adipogenic differentiation [10,11,12].

MSCs were initially studied for their ability to support hematopoietic stem cells in the bone marrow [4,5]. However, they are now being recognized for their regenerative and immunomodulatory properties, as they home into injured tissues and contribute to tissue repair and suppression of inflammatory damage [13,14,15]. In particular, MSCs can sense inflammatory signals and adopt a pro- or anti-inflammatory phenotype to modulate innate and adaptive immunity [15]. For instance, MSCs suppress the proliferation of CD4+ T cells, CD8+ T cells, while promoting the generation of myeloid-derived suppressors cells (MDSC) that further increase local and systemic immunosuppression [16,17,18]. Moreover, MSC may also infiltrate the tumor microenvironment, contribute to tumor growth, and increase resistance to chemotherapy [19,20].

Chemotherapy, beyond its function in controlling the progression of hematological and solid neoplasms, can influence the function and cellular distribution of BM niches by altering cellular and molecular pathways and modifying transformed cells’ behavior [21,22]. In MSC, chemotherapy may induce myelosuppression, and influences their MSCs biological functions, which leads to complications of bone tissue, such as bone fragility because of the decrease in osteoblasts number [23,24,25,26,27]. Moreover, chemotherapy can promote cellular senescence in non-cancerous cells, including fibroblasts and MSCs, which profoundly influence their biological functions [28,29].

We previously demonstrated that HU induces a senescence-like phenotype in peripheral blood MSCs, which reduces its capacity to support human erythroleukemia cell proliferation in vitro [30]. However, whether HU influences the inherent biological MSCs roles is not well elucidated so far. In this study, we investigated if HU may alter human BMMSC differentiation and immunosuppressive functions. Our results suggested that HU-induced BMMSC senescence, including cell proliferation arrest, reducing cell differentiation while increasing immunosuppressive activity on immune T-cell likely via downregulation of mTOR signaling.

## 2. Materials and Methods

Reagents: The following reagents were used: Anti-CDKN2A/p16^INK4A^ antibody (1D7D2A), obtained from Abcam (Cambridge, UK). Anti-p21^Cip1^ was provided by DakoCytomation A/S (Glostrup, DK). Anti-β-actin was from R&D Systems (Minneapolis, MN, USA). Antibodies against phospho-ERK1/2T202/Y204), phospho-p38 and p38, phospho-mTOR and mTOR were purchased from Cell Signaling Technology Inc. (Beverly, MA, USA). HU, CFSE, propidium iodide (PI), Diamidine-2′-phenylindole dihydrochloride (DAPI), Dimethylthiazol-2-yl-2,5-diphenyltetrazolium bromide (MTT), doxorubicin, and Everolimus were provided by Sigma-Aldrich Chemie GmbH (Minneapolis, MN, USA).

Cells and culture conditions: Human MSCs were isolated from the iliac crest bone marrow of healthy voluntary donors and isolated as published previously [31] and cultured in a-Modified Eagle Medium supplemented with 10% fetal bovine serum (FBS) (Sigma-Aldrich Chemie GmbH, Minneapolis, MN, USA). Cells were then propagated until passage three and used for characterization by flow cytometry. Exponentially growing cells passage 3 to 8, were used for indicated experiments. PBMSC were isolated as is described in [30].

Informed consent was obtained from all of the participants included in the study, which has been approved by the Ethical Committee of the Institute for Medical Research, University of Belgrade. MSCs derived from three different donors were used in this study (Age average: 45 years old).

HS-5 (CRL-11882™) cells were purchased from the ATCC (Manassas, VA, USA) and were grown in Dulbecco’s Modified Eagle Medium (SIGMA-Aldrich, Minneapolis, MN, USA) supplemented with 10% FBS. Cells were maintained at 37 °C in a humidified incubator with 5% CO_2_ and medium was replaced every 2–3 days. 

Mesenchymal stem cells characterization: To perform the MSCs immunophenotyping, cells at third-passage were stained with fluorescent dye-conjugated antibodies specific for the following human cell-surface antigens: CD11b-PE, CD45-FITC, CD73-APC, CD90-PE, CD105-FITC, (Biosource, Thermo Fisher Scientific, Waltham, MA, USA). Immuno-labelled cells were evaluated using CyFlow CL (Partec, Munster, Germany). For each sample, at least 10,000 events were recorded.

BM-MSC differentiation

To determine the BMMSCs’ multipotency capacity, the differentiation into osteocyte and adipocyte lineages were assessed in the presence or absence of 200 µM HU. Of note, HU was added in the induction medium and at the onset of differentiation and replaced with fresh HU every 2 days. For osteogenic differentiation, cells (5 × 10^4^ cells/well) were seeded in 24-well plates and cultured with a specific induction medium (50 mM ascorbic acid, 10 mM β-glycerophosphate, 100 nM dexamethasone in DMEM/10% FBS, (all reagents obtained from Sigma-Aldrich Chemie GmbH, Germany) with or without HU for three weeks. The osteogenic differentiation was assessed by inverted light microscope equipped with a digital camera (model, company) after Alizarin red staining. 

Adipogenic differentiation of BMMSC was induced by culturing the cells in adipogenic induction medium (500 µM isobutyl-methylxanthine, 500 nM dexamethasone, 50 µM indomethacin, 1 µM rosiglitazone, and 0.1 UI/mL insulin diluted in DMEM/10% FB (all obtained from Sigma-Aldrich Chemie GmbH, Minneapolis, MN, USA) with or without HU for three weeks. Cells were then subjected to Oil-Red staining assay, photographed with an inverted light microscope and densitometry analyses were performed by using J-image software (https://imagej.nih.gov, accessed on 20 November 2020) of at least five microphotographs.

Viability assays: 

Cellular viability after antimetabolite treatment was assessed by the MTT assay. Briefly, 5 × 10^3^ cells per well were plated in a 96-well plate 24 h before treatment with 0, 25, 50, 100, 200, 400 and 600 uM HU. Then, cells were allowed to proliferate for 96 h. Following 4 h incubation with 10 μL of 5 mg/mL MTT solution (Sigma-Aldrich Chemie GmbH, Minneapolis, MN, USA) at 37 °C, supernatants were discarded, and precipitated formazan crystals were dissolved with 100 mL of DMSO: Isopropanol (2:3) solution for 20 min at room temperature. Finally, absorbance was measured at 620 nm using a microplate reader [30].

Cell cycle and apoptosis analysis: Cell cycle phase distribution was determined by staining cells with PI. Cultured cells were treated with 200 µM HU for 72 h, harvested, fixed with 70% ethanol for 30 min on ice, and stained with propidium iodide (PI) (50 μg/mL in PBS). DNA contents were examined using a BD FACS Calibur (BD Bioscience, San Diego, CA, USA) and cell cycle phase distribution was analysed using Modfit lt 5 program (Verity Software House, Topsham, ME, USA). Apoptosis was assessed by flow cytometry analysis using the Annexin V–fluorescein isothiocyanate and PI (BD Pharmingen, San Diego, CA, USA) according to the manufacturer’s instructions. Flow cytometry analysis was performed using a FACS-Calibur cytometer and Cell Quest software (Becton Dickinson, Heidelberg, Germany).

Immunofluorescence assay: 10^5^ cells were seeded on a rounded coverslip in 24 well plates, and 24 h later, cells were treated with 200 µM for 72 h. Then, cells were fixed with 4% paraformaldehyde in PBS and permeabilized with 0.2% Triton X-100 for 5 min. Cell monolayers were incubated with anti-p16^INK4^ or -p21^Cip1^, followed by incubation with FITC-labelled secondary antibody and 1 μg/mL DAPI. Finally, cells were mounted with DABCO-Mowiol and photographed using an epifluorescence microscope [30].

Senescence-associated-β-galactosidase assay: Senescence-associated b-galactosidase (SA-β-gal) activity was detected by using Senescence Cells Histochemical Staining Kit (Sigma-Aldrich Chemie GmbH, Germany) according to manufacturer’s instructions. BMMSC were seeded to 50% confluence and treated with 200 µM HU for 72 h. SA-β-gal positive blue-green-stained cells were recorded under an inverted light microscope equipped with a digital camera, and at least five randomly chosen fields were counted to determine the percentage of β-gal positive cells.

Acidic vesicular organelles staining: Acidic vesicular organelles (AVOs) were analysed by vital staining with acridine orange (AO) (Sigma-Aldrich Chemie GmbH, Germany). Acridine Orange is a cell-permeable fluorophore that can be protonated, trapped, and emitting orange fluorescence in acidic vesicular organelles such as lysosomes [32,33]. Briefly, BMMSC treated with 200 µM HU for 48 h and control cells were incubated with AO (10 mg/mL, 30 min at 37 °C). Then, cells were carefully washed three times with 37 °C pre-warmed PBS and DMEM was added. Fluorescence microphotographs were captured using a fluorescence inverted microscope equipped with a digital camera (model, company).

T-cell proliferation suppression assay: BMMSCs, previously treated for 72 h with 200 µM HU and control samples, were cocultured for five days with 1 × 10^5^ CFSE labelled human peripheral blood mononuclear cells (PBMCs) in RPMI supplemented with 10% FCS. T cells were activated using αCD3/CD28 micro-beads (Invitrogen, UK) at a concentration of 3 μL/10^6^ T cells. At the end of the coculture period, PBMCs were harvested and fixed with 2% formaldehyde in PBS, and cell proliferation was determined by monitoring CFSE dilution using a BD FACS-Calibur (Becton Dickinson, Heidelberg, Germany).

Myeloid-derived suppressors cells induction: PBMCs were isolated from healthy volunteer donors after density gradient centrifugation on Lymphoprep. MSC previously treated with 200 µM HU for 72 h and control cells were cocultured with PBMCs (ratio 1:5) for five days. Then, PBMCs were collected and subjected to MDSC immune characterization. Monocytic (M)-MDSC subsets were detected by flow cytometry as HLA-DR^low/−^CD33^+^CD14^+^ CD15^−^. Stained cells were analysed by flow cytometry using BD FACS-Calibur (BD Bioscience, San Diego, CA, USA). For each sample, at least 10 000 events were recorded.

Western blot assay: PBMSC were treated for 4, 6, 24, 48, and 72 h with 200 µM HU, whileBMMSC and HS5 cells were treated with 200 µM HU for 72 h. Cell monolayers were lysed for 20 min at 4 °C in 200 μL of lysis buffer (1% NP-40, 0.5% Triton-X100 in PBS plus 1 mM Na_3_VO_4_, 10 mM NaF, 10 mM EDTA, and protease inhibitors). Next, protein samples were subjected to SDS-PAGE and electrotransferred to Hybond nitrocellulose membranes (GE Healthcare). Saturated membranes with 4% non-fat milk, 0.5% Tween 20 in PBS were incubated with specific primary antibodies, and afterward horseradish peroxidase-conjugated secondary antibodies to (Sigma-Aldrich Chemie GmbH, Minneapolis, MN, USA). Finally, membranes were exposed to an enhanced chemiluminescence detection system and protein band levels were quantified using ChemiDoc Imager and ImageLab software (Bio-Rad, Hercules, CA, USA). PBMSC, BMMSC and HS5 cells were used for Western blot analysis of phospho-mTOR, mTOR, and β-actin, while BMMSC cells were additionally tested for the expression of phospho-ERK, ERK, phosphor-p38 and p38 proteins.

Statistical analysis: Data are given as means (±SEM) from at least three independent experiments. The student’s *t*-test was performed to evaluate the probability of significant differences among the samples with *p* < 0.05 (*) and *p* < 0.005 (**) considered significant.

## 3. Results

### 3.1. Bone Marrow Mesenchymal-Stromal/Stem Cells Characterization 

**Figure 1:**
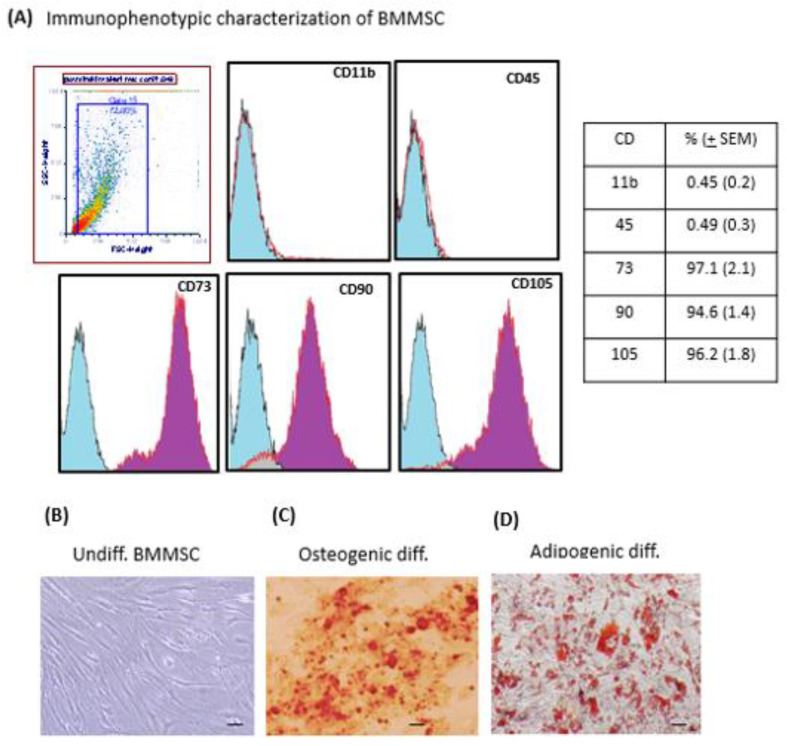
Bone marrow Mesenchymal Stem/Stromal cells characterization (**A**) BMMSC, were subjected to immunophenotyping. Analysis of flow cytometry histogram of BMMSCs for mesenchymal stem cell (CD90, CD73, and CD105) and leukocyte (CD11b and CD45) markers are indicated. Blue light areas indicate background fluorescence obtained with isotype control, and the red-line histogram indicates signal for each specific antibody. Table indicated the average values for the determined cell surface markers. Results presented are representative from at least 3 experiments performed. (**B**) morphology of undifferentiated BMMSC (**C**) osteogenic differentiation determined by alizarin red staining for extracellular matrix mineralization; (**D**) adipogenic differentiation analysed by oil-red staining for cytoplasmic lipid drops accumulation. Magnification 40×, Bars = 10 μm. Results presented are representative from at least 3 performed experiments.

Flow cytometry analysis indicated that isolated BMMSC highly expressed MSC-specific markers CD105 (96.2%), CD73 (97.1%), and CD90 (94.6%), while being negative for hematopoietic stem cell markers such as CD11b (0.45%) and CD45 (0.49%) (Figure 1A). Moreover, cells showed an ability to adhere to plastic and displayed a spindle-like morphology with long cytoplasmic processes (Figure 1B). Furthermore, cells were able to differentiate into osteocytes (Figure 1C) or adipocytes (Figure 1D) under a specific induction culture medium. Together, these results confirmed the MSCs phenotype of cells isolated from the bone marrow.

### 3.2. Hydroxyurea Reduces Bone Marrow Mesenchymal-Stromal/Stem Cell Proliferation 

**Figure 2:**
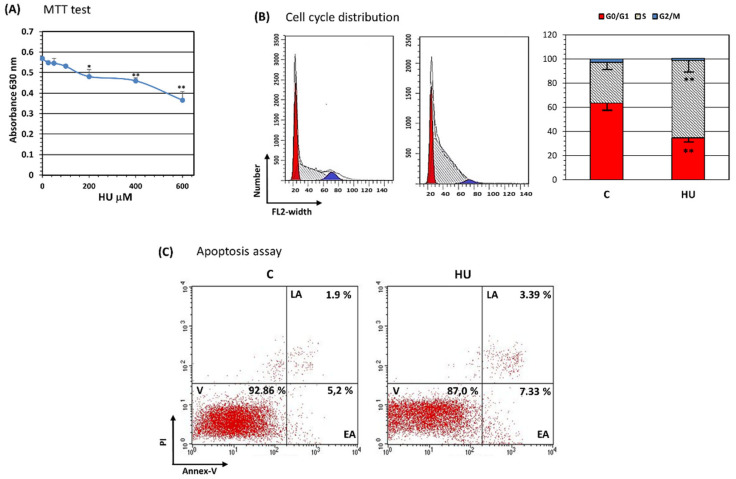
Hydroxyurea inhibits BMMSC proliferation without reducing cell viability. BMMSC were treated for three days with HU and (**A**) proliferation determined by MTT assay. (**B**) Cell cycle analysis was performed by flow cytometry. Results indicated that HU provoked a cell cycle arrest at S phase. (**C**) viability/apoptosis analysis was performed. No differences were obtained compared with control cells. PI, propidium iodide; Annex-V, annexin-V; V, Viable cells; EP, early apoptosis; LP, late apoptosis. Results presented are representative from at least 3 performed experiments. Significant difference control cells by *t*-test: *p* < 0.05 (*) and *p* < 0.005 (**).

HU possesses cytoreduction properties through its capacity to inhibit cell proliferation, mainly by acting as an antimetabolite compound [1]. BMMSC treated with HU concentrations of 200, 400, or 600 µM suffered an increasing inhibition of proliferation as determined by MTT assay (Figure 2A). Because patients’ treatment with HU reaches a blood drug concentration of about 200 µM [34], we decided to use this clinically relevant drug concentration in the following experiments. BMMSC insulted with HU for three days showed a dramatic accumulation in the S phase of the cell cycle, mainly to the detriment of G_0_/G_1_ phase (Figure 2B). These effects on cell proliferation were not accompanied by inducing cell apoptosis; as demonstrated by Annexin-V/PI apoptosis assay, HU-treated BMMSC did not exhibit major differences in apoptosis compared to untreated cells (Figure 2C).

### 3.3. Hydroxyurea Induces Bone Marrow Mesenchymal-Stromal/Stem Cells Senescence 

**Figure 3:**
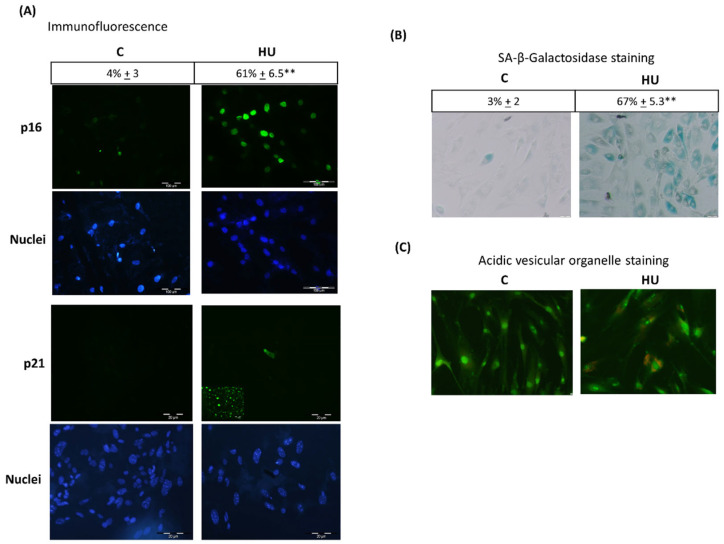
Hydroxyurea induces BMMSC senescence. BMMSC were treated for three days with 200 μM of HU (**A**) fixed and immunostained for p16INK4, magnification 160×, Bars = 100 μm, or p21Cip1, magnification 240×, Bars = 20 μm. Corresponding nuclei were stained with DAPI. P21 Cip1 insert, positive control of BMMSC treated with 100 nM of doxorubicin. (**B**) Subjected to histochemistry analysis for SA-β-gal observed as blue-green cell staining. Magnification 80×, Bars= 10 μm. (**C**) subjected for acidic vesicular organelles (AVOs) staining with acridine orange. Magnification 80×, Bars = 10 μm. Results presented are representative from at least 3 performed experiments. Significant difference control cells by *t*-test, *p* < 0.005 (**).

Previous studies from our laboratory and other authors demonstrated that HU-induced proliferation inhibition triggers cell MSC senescence [30,35]. Next, we analysed the HU capacity to induce p16^ink4^ and SA-β-gal expression in BMMSC, both hallmarks of cell senescence phenotype [36]. BMMSC treated for three days with HU expressed increased immunofluorescence positivity to p16^ink4^ (Figure 3A). Nevertheless, HU-treated cells did not express P21^CIP1^; inserted microphotograph indicates positive control for p21^CIP^ expression after treatment of BMMSC with doxorubicin (100 nM). Furthermore, three-day HU treatment-induced SA-β-gal expression in BMMSC indicated by blue-green staining (Figure 3B). Interestingly, increased SA-β-gal expression has been associated with lysosome increase in number and size [37]. HU treatment resulted in the appearance of AVOs when the cells were stained with acridine orange after 48 h of HU treatment (Figure 3C). 

### 3.4. Hydroxyurea Did Not Influence Surface Marker Expression, Whereas It Reduced Mesenchymal-Stromal/Stem Cells Differentiation

**Figure 4:**
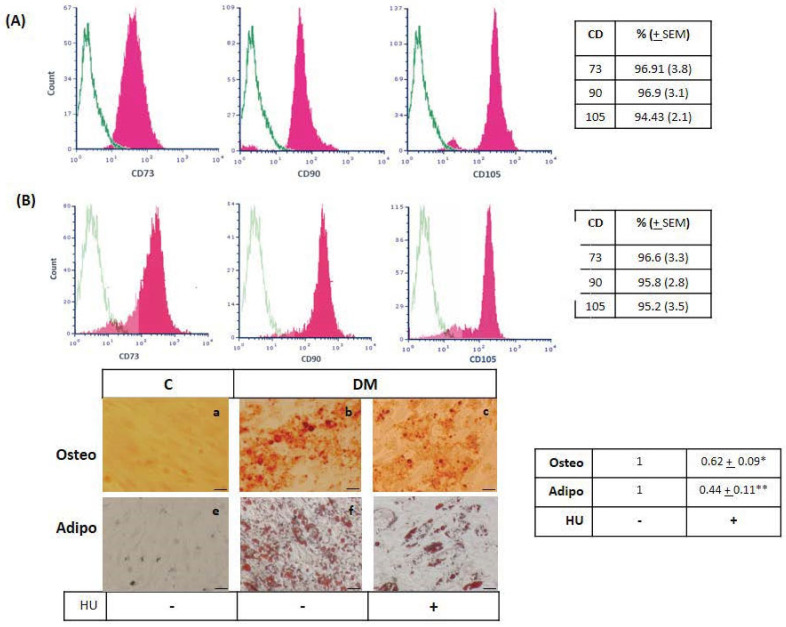
Hydroxyurea inhibits BMMSC differentiation. BM MSC were cultured for 3 days without (**A**) or with (**B**) 200 μM HU and subjected to MSC CD73, CD90 and CD105 surface markers determination by flow cytometry analysis. Green line indicated fluorescence obtained with isotype control. (**C**) Subjected to osteogenic (Osteo) and adipogenic (Adipo) differentiation and determined by alizarin red and oil-red staining respectively. (**a**,**e**) non-differentiated control cells, (**b**,**f**) osteogenic differentiation, and (**c**,**g**) adipogenic differentiation. Table indicates the differentiation level compared to BMMSC without HU treatment (value = 1). Magnification 40×, Bars = 10 μm. Results presented are representative from at least 3 performed experiments. Significant difference control cells by *t*-test: *p* < 0.05 (*) and *p* < 0.005 (**).

Since HU inhibition of cell proliferation is accompanied by a senescent phenotype, we next wondered if drug treatment may alter BMMSC stemness properties. We therefore investigated the expression of conventional BMMSC surface markers by flow cytometry after three days of HU treatment, MSCs specific markers CD73, CD90, and CD105, were expressed to the similar extent as in (Figure 4B) untreated BMMSC (Figure 4A). As the capacity of BMMSC to differentiate under specific stimuli is their defining stemness property, we investigated whether HU may affect osteogenic and adipogenic differentiation. Decreased calcium deposition on BMMSC monolayers, determined by Alizarin red assay, showed that three days HU treatment significantly reduced osteogenic BMMSC differentiation (Figure 4C). Similarly, adipogenesis was also reduced upon HU treatment, observed by decreased intracellular lipid droplet accumulation after oil red O staining (Figure 4C). Thus, despite maintaining the expression of characteristic stem cell markers, HU-treated BMMSC exhibit reduced differentiation potential compared to untreated cells.

### 3.5. Hydroxyurea Enhances Bone Marrow Mesenchymal-Stromal/Stem Cells Immunosuppression

**Figure 5:**
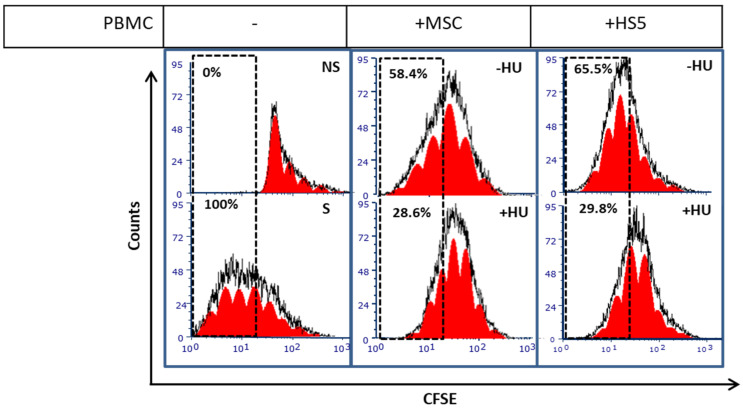
Hydroxyurea enhances the capacity of BMMSC to inhibit T-cell proliferation. BMMSC and HS-5 cell line were pre-treated for three days with 200 μM of HU and subjected to co-culture conditions with CFSE-stained peripheral blood mononuclear cells stimulated with αCD3/CD28 micro-beads for five days. Then, CFSE cellular dilution indicating cellular duplication was analysed by flow cytometry. Experimental control without MSC refers to CFSE-stained PBMC alone under non-stimulated (NS) or stimulated (S) with αCD3/CD28 conditions. Results presented are representative from at least 3 performed experiments.

Next, we wanted to analyse if HU treatment affects BMMSC functionality. Of note, BMMSCs possess a capacity to reduce T-cell proliferation and activation [38]. To test the effect of HU on BMMSC immunosuppression, we pre-treated BMMSC, or HS-5 bone marrow stromal cells with HU for 3 days, then co-cultured them with PBMC in which T-cell proliferation was activated using αCD3/CD28 micro-beads. Untreated BMMSC and HS-5 cells showed basal inhibitory effect on T-cell proliferation by decreasing the proliferation rate to 65.5% and 58.4%, respectively, compared to a 100% proliferation measured in stimulated PBMCs grown in the absence of MSCs (Figure 5). Importantly, HUpre-treated BMMSC and HS-5 cells, exert an increased capacity to inhibit PBMC proliferation compared to untreated, decreasing it to 29.8% and 28.6%, respectively (Figure 5). HU treatment doubles MSC inhibitory capabilities on T-cell proliferation.

### 3.6. Hydroxyurea Inhibits mTOR Signaling in Bone Marrow Mesenchymal-Stromal/Stem Cells

**Figure 6:**
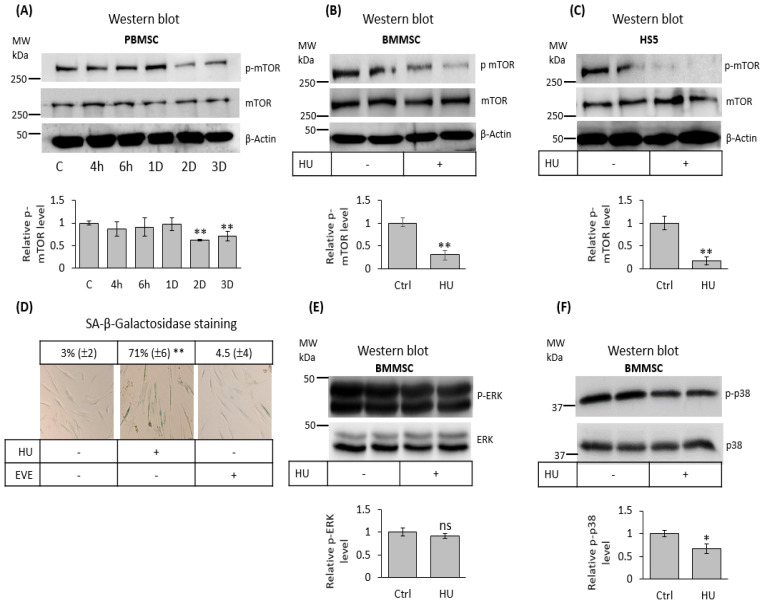
Hydroxyurea inhibits mTOR activation in MSC. (**A**) PBMSC were treated for the indicated period of time with 200 μM HU and subjected to mTOR phosphorylation analysis by Western blot. (**B**,**C**) BMMSC and HS-5 cell line treated for three days with 200 μM of HU and subjected to mTOR phosphorylation analysis by Western blot. Top tables indicated the relative densitometry values for the obtained signals. α-actin is determined as loading control. (**D**) BMMSC were three days treated with 200 μM of HU or 100 nM Everolimus (EVE) and subjected to SA-β-gal histochemistry assay. (**E**,**F**) BMMSC and HS-5 cell line were treated for three days with 200 μM of HU and subjected for ERK1,2 and p38 MAPK phosphorylation analysis by Western blot assay. *n* = 3, mean ± SEM, *p* < 0.05 (*) and *p* < 0.005 (**).

Inhibition of cell proliferation occurs due to decreased proliferative extracellular and intracellular signaling, resulting in cell cycle arrest [39,40]. We therefore tested if HU treatment alters the activation of different signalling pathways in MSC. PBMSC subjected to HU treatments lasting from 4 h to three days showed that a reduction of mTOR phosphorylation/activation occurs with longer HU exposure of 48–72 h (Figure 6A). mTOR inhibition was further confirmed after three days of HU treatment of BMMSC and HS-5 cells (Figure 6B,C). Nevertheless, mTOR inhibition by 100 nM of Everolimus did not induce BMMSC senescence as determined by SA-b-gal staining (Figure 6D). In addition, HU did not substantially modify ERK1/2 phosphorylation of BMMSC after three treatment days (Figure 6E), while it slightly reduced the expression of downstream phospho-p38, indicating possible involvement of ERK/MAPK signalling pathway (Figure 6F).

### 3.7. Hydroxyurea Did Not Alter Bone Marrow Mesenchymal-Stromal/Stem Cells’ Induction of Myeloid-Derived Suppressor Cells 

**Figure 7:**
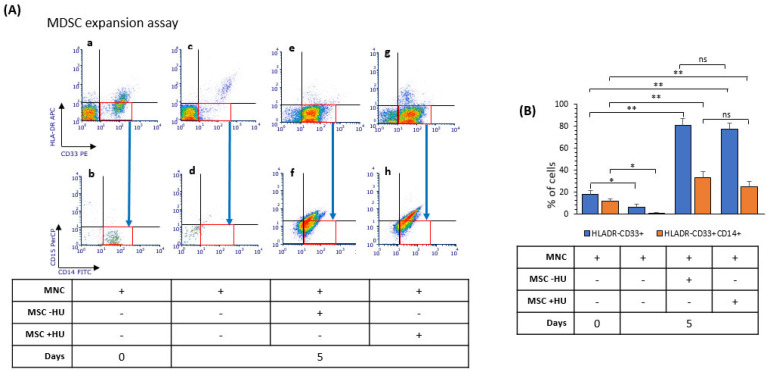
Hydroxyurea does not alter the capacity of BMMSC to induce monocytic myeloid-derived suppressor cells expansion. (**A**) BMMSC were pre-treated for three days with 200 μM of HU and co-cultured with-PBMC isolated from healthy donors for five days. Then, PBMC were subjected to immunostaining for HLADR, CD33, CD14, CD15 surface antigens by flow cytometry. Gating was performed to distinguish to populations of cells HLADR-CD33+ cells and M-MDSC (HLADR-, CD33+, CD14+, CD15-) Red squares indicate the selected gating areas for quantification analysis. (**B**) Bar plot for HLADR-CD33+ cells and M-MDSC quantification. Results presented are representative from at least 3 performed experiments. Significant difference control cells by *t*-test: *p* < 0.05 (*) and *p* < 0.005 (**).

MSCs further contribute to immunosuppression by inducing MDSC expansion and function [41,42]. Next, we investigated whether HU may influence the capacity of BMMSC to induce the expansion of MDSC. Consequently, we cultured PBMC isolated from healthy subjects with untreated MSC or MSC treated with HU. After five days, the amount of M-MDSC was analysed based on the expression of specific surface markers CD33^+^HLA-DR^-^CD14^+^ by flow cytometry. Both control BMMSCs and HU-BMMSCs induce similar M-MDSCs number (Figure 7). Comparable results were observed by co-cultivating HS-5 cells with or without HU pre-treatment (data not shown). While having a strong inhibitory effect on T-cell proliferation, HU-treated BMMSC do not influence M-MDSC expansion.

## 4. Discussion

HU is a water-soluble antiproliferative agent used in neoplastic and non-neoplastic conditions, such as hematological malignancies, infectious diseases, and dermatology for refractory psoriasis [1]. Mainly, HU arrests cells in the S phase of the cell cycle due to the decrease of dNTPs pools resulting from RNR inhibition and a slowdown of the DNA polymerase movement at replication forks [1,43]. Beyond cancer cells, non-transformed cells are also susceptible to the profound influence of chemotherapy [44,45]. Here, we aimed to determine the main aspects of the HU treatment on BMMSC functionality. BMMSC play role in different aspect of cellular and tissue biology, such as tissue regeneration, differentiation, immunomodulation, and as part of the hematopoietic niches [5,46]. Moreover, the BMMSC may infiltrate the tumor microenvironment supporting cancer cell growth, activate mitogen and stress signaling, as well as increase resistance to chemotherapy [19]. Our results indicated that HU effectively reduce BMMSC proliferation with no effect on cell viability, which is in concordance with the notion of that HU acts mainly as a cytostatic agent [1,43]. 

Cellular senescence is defined as irreversible proliferative cell arrest with secretory features that contributes to aging and age-related diseases [47]. HU belong to the family of anti-metabolite anticancer-drugs, which provoke biological stress and lead to cellular senescence in both transformed and non-transformed cells, in a process defined as chemotherapy-induced senescence [48]. Our results indicate that the BMMSC, under HU treatment, display a senescence phenotype as observed by p16INK4 expression and SA-β-gal positivity alongside with lysosome alterations. Interestingly, HU did not provoke P21CIP expression in BMMSC, similarly to HU-induced senescence in peripheral blood-derived MSC [30]. This is in accordance with literature data showing that HU acts as a replication stress inducer, which alters epigenetic regulation of p16 CDK inhibitor expression, while P21CIP expression depends on DNA damage-induced cellular senescence [28]. Nevertheless, relatively high HU concentration may induce p21CIP stabilization without de novo synthesis in a human embryonic fibroblast cell line or induce p53-p21CIP axis in foreskin fibroblast [34,49,50]. Thus, this reinforces the concept of the HU mechanism of senescence depending on the cell type, as well as drug concentration and time of exposure. 

MSC play crucial roles in tissue regeneration due to their inherent capacity to differentiate towards different lineages [51]. However, senescent MSC may exhibit, parallel to proliferation arrest, dysfunctional capacity of differentiation, in part due to the profound changes in protein expression and chromosome structure [52,53]. Our results indicated that HU treatment reduced osteogenic and adipogenic differentiation capacity of MSC, without affecting the expression of cell surface markers (Figure 4). Similar results have been reported in dental follicle-derived MSC, where the proliferation and clone formation capacity were impaired by HU accompanied by reduced MSC differentiation toward adipogenic, chondrogenic, and osteogenic lineages [54]. Conversely, treatment with other antimetabolite drugs—namely, 5-FU and gemcitabine—retain BMMSC multi-lineage differentiation potential, although no senescence analysis was performed in this study [55]. These data suggest that chemotherapy does not modify MSC differentiation markers, thus MSC may preserve their stem identity. 

Besides differentiation, MSC also play a role in immune response and inflammation. MSC strongly regulate lymphocyte T-cell proliferation and activation [56,57], which may significantly influence immune surveillance within a tumor microenvironment. In our study, HU greatly enhanced the capacity of BMMSC to inhibit T-cell proliferation (Figure 5). When MSC undergo senescence, they remain metabolically active considerably alter the composition of secretome to senescence-associated secretory phenotype (SASP); transforming growth factor (TGF)-β is one of the members of SAPs that regulates immune response and strongly inhibit T-cell activation and function [58,59]. HU-induced MSC senescence is accompanied with increased TGF-β expression [30], which in part may explain the improved BMMSC capacity to inhibit T-cell proliferation. In addition, senescent mesenchymal-derived cells also produce elevated levels of reactive oxygen species (ROS) [30,60], which may contribute to SASP and suppression of T-cell function [61,62]. Nevertheless, further analyses are necessary to elucidate the exact contribution of TGF-β and ROS in the increased capacity of HU-induced MSC senescence.

Interestingly, mTOR signaling plays crucial roles in senescence establishment. This kinase participates in the conversion of cell arrest to senescence phenotype, also called geroconversion, which consider cell cycle inhibition by p16 or p21 and active mTOR and ERK1,2 MAPK signaling driving geroconversion towards senescence [63]. Thereby, active mTOR seems to be necessary for the induction of cellular senescence [64,65]. Conversely, our results show that HU-induced MSC senescence provoked a reduction of mTOR activation (Figure 6 A–C). Furthermore, the inhibition of mTOR by Everolimus did not induce BMMSC senescence (Figure 6D), thus suggesting that mTOR inhibition, although it can be necessary, is not sufficient to induce cellular senescence in our experimental conditions. In fact, mTOR inhibition by rapalogs, such as Everolimus, function as a senolytic event with potential use in anti-aging strategies [66]. Moreover, mTOR inhibition prevents the permanent proliferation arrest converting it into a reversible condition [67]. Nonetheless, it is necessary in new experiments to determine the precise mechanism involved in mTOR downregulation and whether this signaling plays roles in pre-senescent and senescent stages of MSC under HU treatment.

Besides, mTOR signaling tightly regulates the differentiation of MSC reviewed in [10], which may explain decreased differentiation capacity of senescent BMMSC towards osteocyte and adipocyte lineages. Furthermore, mTOR inhibition contributes to an enhanced capacity of MSC to inhibit T-cell proliferation and activation [68,69]. Thus, it is possible to speculate that mTOR signaling downregulation by HU during the induction of MSC senescence mediates the inhibition of osteogenic and adipogenic differentiation, as well as contributes to the improvement of the immunomodulatory capacity of BMMSC. 

Even though HU did not influence ERK1,2 signaling, it led an inhibition of p38 MAPK that may also contribute to the reduced osteogenic differentiation of senescent BMMSC, since p38 signaling seems to be critical for the committed of MSC toward osteoblastic fate [70]. To date, the underlying molecular mechanism involved in the regulation of intracellular signaling by HU is not well understood, but it has been suggested that HU may promote epigenetic modifications that may affect several intracellular signal transductions, such as MAPK, PKG, and PKA signaling [71,72].

Finally, HU-induced senescent BMMSC preserve their capacity to induce monocytic-MDSC in vitro (Figure 7), indicating that, in part, they capacity to influence myeloid cells is not mainly influenced by HU treatment. Interestingly, the capacity of BMMSC to induce MDSC expansion further contributes to the stabilization of an anti-inflammatory microenvironment that, depending on the disease, may reduce the effectiveness of treatments, such as in cancer, while it may have therapeutic benefits in autoimmune disorders [73].

## 5. Conclusions

HU induces BMMSC senescence alongside reducing cellular differentiation and increasing T-cell immunoregulatory activities, which may be related to inhibition of the mTOR signaling. Our study contributes to the understanding of chemotherapy-induced alterations in bone marrow-derived MSC functionality. Cellular senescence could be a side effect of HU treatment chemotherapy regimens of patients that should be considered in order to avoid harmful treatment-associated comorbidities.

## Data Availability

The data presented in this study are available on request from the corresponding author.
